# MEK inhibition enhances efficacy of bacillus Calmette-Guérin on bladder cancer cells by reducing release of Toll-like receptor 2-activated antimicrobial peptides

**DOI:** 10.18632/oncotarget.18230

**Published:** 2017-05-26

**Authors:** Young Mi Whang, Su Bin Jin, Serk In Park, In Ho Chang

**Affiliations:** ^1^ Department of Urology, Chung-Ang University College of Medicine, Seoul, Korea; ^2^ Department of Biochemistry and Molecular Biology and BK21 Plus Program, Korea University College of Medicine, Seoul, Korea; ^3^ Department of Medicine, Vanderbilt University School of Medicine, Nashville, TN, USA

**Keywords:** bacillus Calmette-Guérin (BCG), Toll-like receptors 2 and 4 (TLR2 and TLR4), antimicrobial peptides (AMPs), MEK inhibitors, bladder cancer cells

## Abstract

Bacillus Calmette-Guérin (BCG) is one of the standard treatment options for non-muscle-invasive bladder cancer. The details of the biological defense mechanisms against BCG remain unclear. Here, we investigated whether BCG-induced release of antimicrobial peptides (AMPs; e.g., human β-defensin-2, -3, and cathelicidin) is involved with mitogen-activated protein kinase (MAPK) pathways, and investigated the enhanced anticancer effect of BCG through the down-regulation of Toll-like receptors (TLRs) and MAPK pathways in bladder cancer cells. BCG-infected bladder cancer cells produced AMPs as a defense mechanism against BCG, which were reduced by MEK inhibitors by blocking phosphorylation of extracellular signal-regulated kinase (ERK1/2 or MEK) and c-Jun. MEK inhibitors enhanced inhibition of bladder cancer cell growth by decreased binding of c-Jun, p65 and Pol II to the activated protein-1 promoter. Knockdown of TLR2 and TLR4 reduced ERK phosphorylation. Knockdown of TLR 2 decreased release of AMPs, which was similar to the efficacy of MEK inhibitor on BCG-infected cells. BCG-infected bladder cancer cells were more prone to induction of AMP release following TLR2 activation via ERK and c-Jun pathway mediators. In conclusion, our data suggest that the BCG-induced release of AMPs in bladder cancer cells is a promising molecular target for enhancing the immunotherapeutic efficacy of BCG in bladder cancer patients.

## INTRODUCTION

Bacillus Calmette-Guérin (BCG) is a potent vaccine for *Mycobacterium tuberculosis*. BCG is one of the most successful immunotherapeutics for cancer, and is included in the standard treatment regimens for high-risk non-muscle-invasive bladder cancer patients [1]. However, BCG is less effective for recurrent or advanced-stage bladder cancer patients [2]. To improve the efficacy of BCG, inhibition of pro-inflammatory mediators in the innate and acquired immune responses against mycobacteria must be clearly understood [3, 4].

During initial recognition of pathogens like BCG mycobacteria, Toll-like receptors 2 and 4 (TLR2 and TLR4, respectively) are activated to elicit immune responses [5]. Activation of TLRs releases antimicrobial peptides (AMPs) and pro-inflammatory cytokines via nuclear factor-κB (NF-κB) pathways [6, 7] and mitogen-activated protein kinases (MAPK) pathways, leading to modulation of transcription of inflammatory genes [8, 9]. The association of TLR2 with TLR1/TLR6 or TLR4 in turn recruits different adaptor proteins allowing specific signaling cascades and gene activation programs, which contribute to resistance against mycobacteria [10]. Accordingly, mycobacterial components act as TLR agonists and elicit the release of pro- or anti-inflammatory cytokines [11].

Mammalian cells secrete numerous AMPs, such as human beta-defensins (HBD-1 or HBD-4) in epithelium and leukocytes, and cathelicidin (CAMP) in neutrophils and epithelia [12]. Mycobacteria trigger epithelial cells to express AMPs. AMPs have nonspecific cytotoxicity against a wide range of normal and malignant targets, and direct lyse mycobacteria by permeablizing the cellular membranes [13, 14]. BCG induces HBD-2 mRNA expression in human lung epithelial cells, which exhibits antimicrobial activity against BCG despite up-regulated tumor necrosis factor-alpha (TNF-α) production by BCG-infected cell [15]. Furthermore, BCG-induced HBD-2 expression affects the internalization rate of BCG in bladder cancer cells, whereas the anti-HBD-2 antibody prevents the effect of HBD-2 on BCG internalization in bladder cancer cells [16]. Therefore, modulation of anti-mycobacterial activity against BCG, such as TLR downstream signaling pathways, will potentially lead to new therapeutic strategies in mycobacteria treatment.

MAPK pathways are crucial to mycobacteria-induced macrophage signaling via TLRs [8, 9]. Similar to the molecular mechanisms by which mycobacteria upregulates AMPs in epithelial cells, MAPK pathway activation contributes to the regulation of inflammatory processes in BCG-infected epithelial cells. HBD-2 participates in anti-bactericidal activities directed against BCG, which is mediated by MAPK signaling pathways regulating HBD-2 expression in human epithelial cells during BCG infection [17]. Furthermore, *M. bovis* BCG-mediated TLR2 signaling triggers the production of nitric oxide, which negatively regulates interferon-gamma (IFN-γ)-induced immune gene expression for macrophages [18].

The present study demonstrates that MEK inhibitors enhance BCG treatment-induced tumor cell death via the blockage of AMPs release. The enhanced antitumor effects of BCG in bladder cancer cells are associated with the inhibition of TLR2-medated MEK pathway. The findings implicate the activation of intracellular signaling pathways in response to BCG infection as a novel strategy to boost BCG treatment efficacy in urothelial carcinomas.

## RESULTS

### BCG stimulates release of AMPs and induce ERK (1/2) phosphorylation in bladder cancer cells

**Figure 1:**
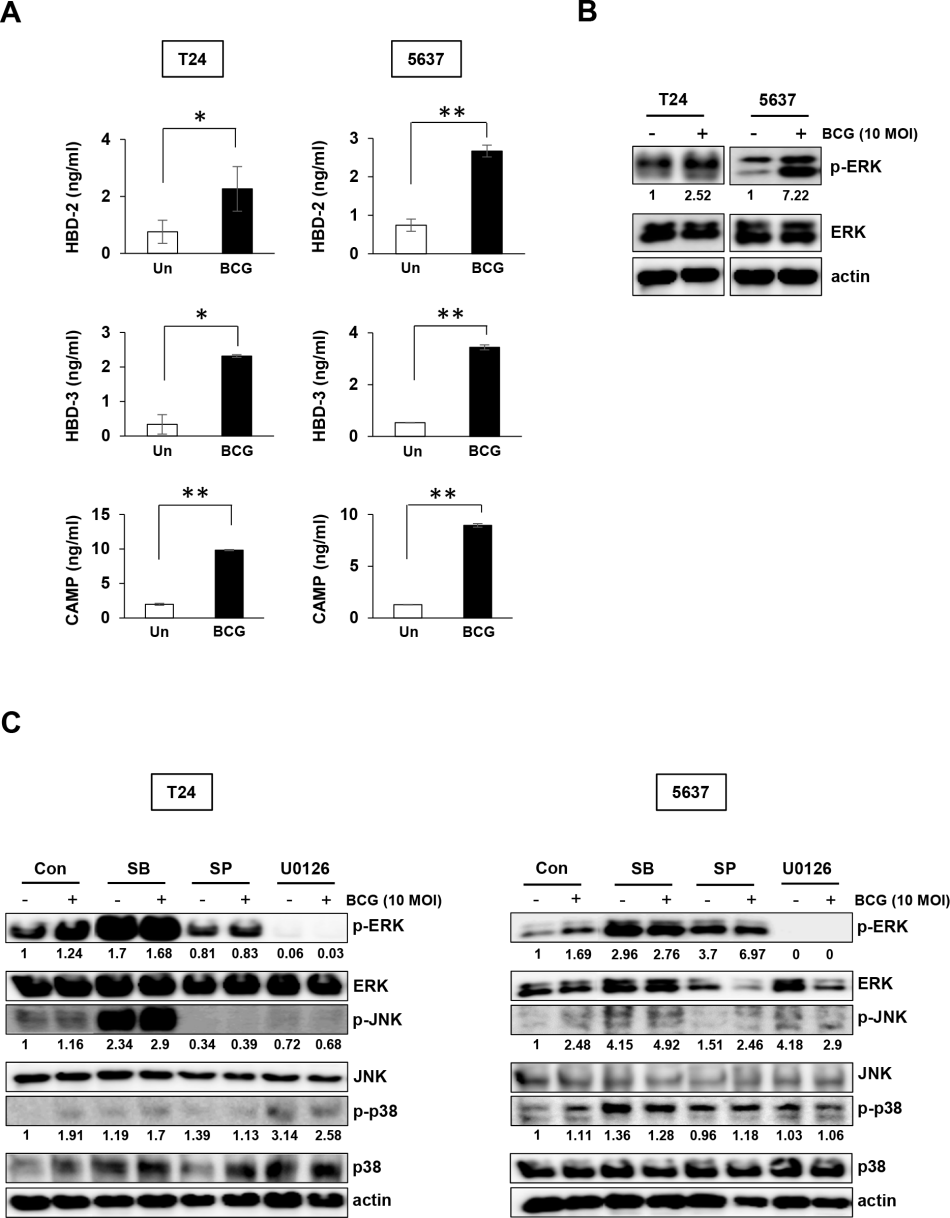
BCG stimulates release of antimicrobial peptides and induces ERK phosphorylation in two bladder cancer cell lines **(A)** T24 and 5637 bladder cancer cells were infected with BCG (10 MOI for 8 h) or empty vector (Un; untreated), followed by ELISA of antimicrobial peptides (HBD-2, HBD-3, and CAMP) in the culture supernatant. Data are mean ± SD (n=3 per group). * *P*<0.05 and ** *P*<0.01, Student's T-test **(B)** Western blotting analyses showing BCG-induced ERK phosphorylation in T24 and 5637 cells. **(C)** Western blotting analyses showing phosphorylation of ERK, JNK, and p38 in T24 (left panels) and 5637 (right panels) cells pre-treated with SB203580 (SB; a specific p38 inhibitor; 10 μM), SP600125 (SP; a specific JNK inhibitor; 5 μM), or U0126 (an MEK1/2 or ERK1/2-specific inhibitor; 10 μM) for 2 h, followed by BCG infection (10 MOI for 8 h). DMSO was used as control (Con). Numerical data represent phospho-ERK/total ERK and phospho-JNK/total JNK ratios determined by densitometric analyses. All expression ratios were normalized to the untreated group.

To determine the effect of BCG-induced AMPs release on bladder cancer cells, the cells were treated with 10 MOI BCG for 8 hours, followed by ELISA quantification of AMPs. BCG stimulated the release of HBD-2 and -3 by 3-fold compared to untreated control in both types of bladder cancer cells. The CAMP level was increased by over 8-10-fold in BCG-treating bladder cancer cells compared to untreated cells (Figure 1A). We hypothesized that BCG-induced expression of inflammatory mediators, including chemokines and AMPs, is associated with the MAPK signaling pathway. Previous reports showed that BCG activates the MAPK and phosphoinositol-3 kinase pathways as signaling events leading to pro-inflammatory gene expression [19, 20]. Therefore, we determined whether BCG-dependent activation of MAPK pathway can be blocked by MAPK-specific inhibitors in bladder cancer cells. ERK phosphorylation was induced by BCG treatment in both 5637 and T24 cells (Figure 1B) and the effect was completely blocked by MEK inhibitor in both 5637 and T24 cells. JNK inhibitors also blocked phosphorylation of JNK only in T24 cells (Figure 1C). These results suggest that BCG treatment can stimulate release of antimicrobial peptide via phosphorylation of ERK in bladder cancer cells.

### BCG treatment selectively induces the MEK pathway in bladder cancer cells

**Figure 2:**
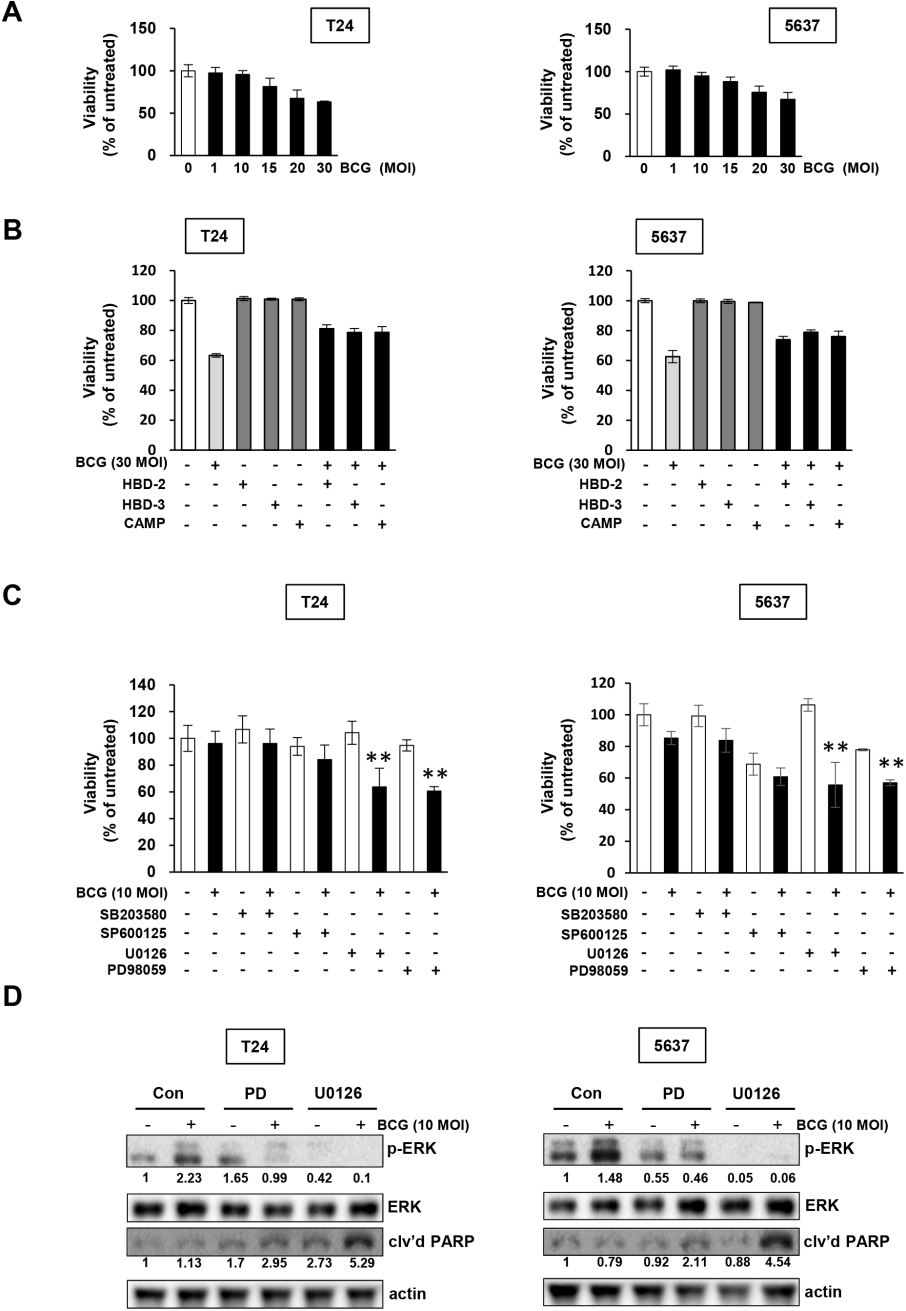
Pharmacological inhibition of MEK pathways increases the anti-proliferative effects of BCG in bladder cancer cells **(A)** Cells were plated in 96-well plates 24 h before adding BCG. Viable cells were quantified by MTT assays. **(B)** Cells were pretreated with antimicrobial peptides (HBD-2, HBD-3, and CAMP), and subsequently infected with 30 MOI of BCG (i.e. a dosage determined by data in panel A to induce greater than 30 % growth inhibition). After 48 hours, viable cells were quantified by MTT assays. **(C)** Cells were pretreated with specific inhibitors of p38 (SB203580; 10 μM), JNK (SP600125; 5 μM) and/or ERK (U0126; 10 μM or PD98059; 1 μM). After 2 hours, the cells were infected with 10 MOI of BCG (i.e. sub-optimal dosage to suppress cell proliferation as shown in the panel A) for 48 hours, followed by MTT cell viability assays. Data are mean ± SD (n=6 per group). **P<0.01, U0126/ BCG+U0126, BCG/BCG+U0126, PD98059/ BCG+PD98059, or BCG/BCG+PD98059. Student's T-test. **(D)** Western blotting analyses showing apoptosis by combined treatment with MEK inhibitors and BCG in T24 (left panels) and 5637 (right panels) cells pre-treated with PD98059 (a specific MEK1 inhibitor; 1 μM) or U0126 (a specific MEK1/2 inhibitor; 10 μM) for 2 h, followed by BCG infection (10 MOI for 48 hours). DMSO was used as empty vector controls (Con). The expressions of phosphorylated ERK1/2 was calculated as the ratio of phosphorylated ERK/2 to total ERK1/2 protein expression using densitometric analyses. The ratio of cleaved PARP product to actin protein expression were calculated similarly. Expression ratios were normalized to the untreated group.

We hypothesized that inhibition of MEK pathway avoids a BCG-induced antimicrobial effect on bladder cancer cells, resulting in blockage of the release of AMPs. We next tested whether BCG treatment in bladder cancer cells in combination with the MAPK specific inhibitors U0126, PD98059, SB20358, or SP600125 more effectively inhibited tumor cell proliferation. Both T24 and 5637 cells exhibited resistance to BCG single treatment, with no growth inhibitory effect by 10 MOI BCG (Figure 2A). Then, we observed that high concentration of 30 MOI BCG induced growth inhibition by 40%, which was reduced by treatment with recombinant AMPs in both T24 and 5637 cells (Figure 2B). Sensitivity to BCG was exacerbated by the U0126 and PD98059 MEK inhibitors, which inhibit MEK1/ MEK2 (U0126 inhibits both MEK1/2; PD98059 inhibits a highly selective MEK1 activation) compared with BCG in combination with other MAPK inhibitors (Figure 2C). These results are consistent with the involvement of the BCG-induced MEK pathway and blockage of phosphorylated ERK by MEK inhibitors (Figure 1B and 1C). As shown in Figure 2D, treatment of BCG with MEK inhibitors abolished phosphorylation of EKR and induced cleavage of poly (ADP-ribose) polymerase (PARP), suggesting that combined treatment with BCG and MEK inhibitors induces apoptosis in BCG-treated bladder cancer cells. Therefore, inhibition of the ERK pathway could increase sensitivity to BCG by repressing BCG-induced phosphorylation of ERK and subsequent inhibition of the release of AMPs.

### MEK inhibitors reduce BCG-mediated transcriptional activation of AMPs in bladder cancer cells

**Figure 3:**
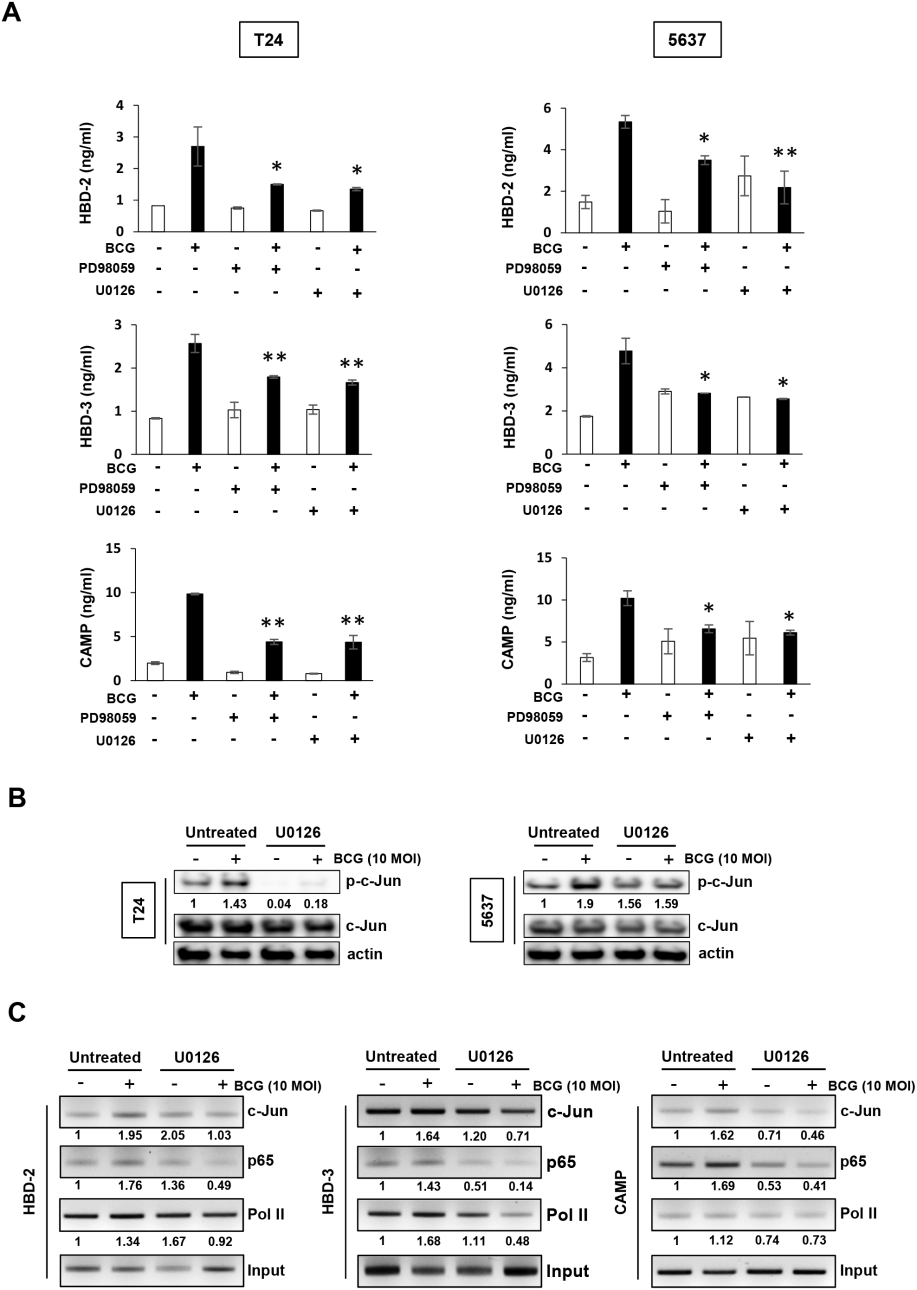
Inhibition of MEK pathway suppresses BCG-mediated release of AMPs in bladder cancer cells **(A)** T24 (left panels) and 5637 (right panels) bladder cancer cells were pretreated with two MEK-specific inhibitors (U0126 10 μM or PD98059 1 μM for 2 h), and subsequently infected with BCG (10 MOI for 8 h). Antimicrobial peptides (HBD-2, HBD-3, and CAMP) in culture supernatants were quantified by ELISA. Data are mean ± SD of 3 independent experiments. *P<0.05 and ** *P*<0.01, BCG/BCG+PD98059 or BCG/BCG+U0126. Student's T-test. **(B)** Western blotting analyses showing BCG-induced c-Jun phosphorylation is suppressed by MEK inhibitors in both T-24 and 5637 cells. Cells were pretreated with an MEK-specific inhibitor (U0126; 10 μM, PD98059; 1 μM) for 2 h. Subsequently, the cells were infected with BCG (10 MOI for 48 h) and cell lysates were subjected to Western blot analysis. Phospho-Jun/total Jun expression ratios were determined by densitometric analyses. Expression ratios were normalized to the untreated group. **(C)** Chromatin immunoprecipitation assay showing that MEK inhibition reduces BCG-mediated AMP release in bladder cancer cells. T24 cells were treated with U0126 (10 μM for 2 hours). The cells were subsequently infected with BCG (10 MOI for 8 hours) and fixed with 10% formaldehyde. The lysates were subject to ChIP assay using anti-c-Jun, -p65, and -Pol II antibodies. Expressions of AMPs were calculated as the ratio of c-Jun, p65, and Pol II to input DNA expression using densitometric analysis. All expression ratios were normalized to the untreated group.

We first evaluated the effect of MEK inhibitors on the reduction of BCG-induced AMPs release from the supernatants of conditioned media. As shown in Figure 3A, in T24 cells, BCG increased in the release of three AMPs, and this effect was suppressed by MEK inhibitors. BCG-induced HBD-2/3 and CAMP release in 5637 cells were reduced by treatment with MEK inhibitors. Given the activation of the transcription factor AP-1 is essential to HBD-2 and -3 and/or CAMP production, we sought to determine the binding of c-Jun, p65, and/or Pol II to the AP1 promoter in BCG- and/or MEK inhibitor-treated T24 cells using a ChIP assay. Phosphorylation of c-Jun was increased in BCG-treated bladder cancer cells, but BCG-induced phosphorylation of c-Jun was completely abolished by MEK inhibitor in T24 cells (Figure 3B). In 5637 cells, BCG-induced phosphorylation of c-Jun was reduced in comparison to BCG alone treatment. This effect was involved in activation of ERK phosphorylation, which was reduced by treatment with MEK inhibitors (Figure 2D). Therefore, we next tested whether c-Jun, p65, and Pol II binding to the HBD-2, -3 or CAMP promoters could be increased by BCG treatment. As shown in Figure 3C, following treatment with MEK inhibitors, binding of c-Jun and p65 to the HBD-2 promoter was reduced in T24 cells, but the binding of Pol II was only slightly reduced. Inhibition of c-Jun binding to HBD-3 by MEK inhibitors was less efficient than p65 and Pol II, but c-Jun and p65 binding to CAMP promoter was strongly reduced by MEK inhibitor in comparison with Pol II. These data collectively indicate that BCG induces transcriptional activity of AMPs, which can be blocked by MEK inhibitor in bladder cancer cells, and which is potentially mediated by recruitment of AP-1 subunit c-Jun, p65, and Pol II.

### BCG-induced AMPs release is mediated by TLR2 in bladder cancer cells

**Figure 4:**
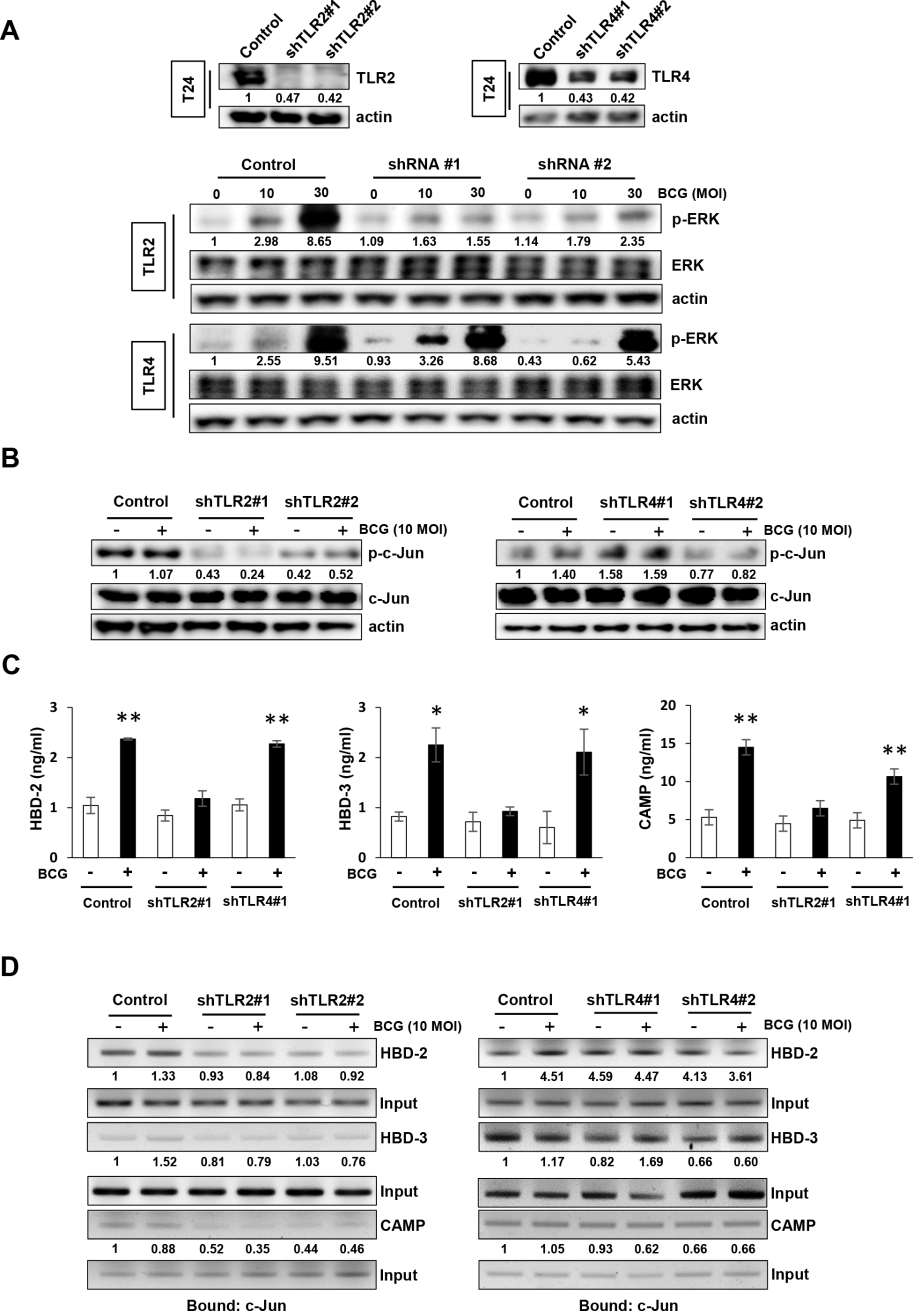
TLR2 participates in BCG-induced activation of MEK pathway for AMP release in bladder cancer cells **(A)** Western blotting analyses demonstrating the reduction of Toll-like Receptor 2 (TLR2) or TLR4 protein expression in T24 cells transfected with two shRNA-expressing plasmids targeting TLR2 or TLR4 sequences (shTLR2#1, shTLR2#2, shTLR4#1 and shTLR4#2, respectively; upper panels). Non-specific scrambled shRNA was used as control. shRNA TLR2 or TLR4 knockdown cells were selected with puromycin, and infected with BCG (10 or 30 MOI for 48 h; lower panels). Phospho ERK expression was detected by Western blotting. **(B)** Stable clones of TLR2- or TLR4-knockdown T24 cells were treated with BCG (10 MOI for 8 h) and analyzed for expression of phosphorylated c-Jun. Phospho-Jun/total Jun expression ratios were calculated by densitometric analysis, and indicated under each lane. Expression ratios were normalized to the untreated group. **(C)** StableshTLR2 or shTLR4 T24 bladder cancer cells were infected with BCG (10 MOI for 8 h), followed by ELISA of antimicrobial peptides (HBD-2, HBD-3, and CAMP) in the culture supernatant. Data are mean ± SD (n=3 per group). * *P*<0.05 and ** *P*<0.01 Student's T-test. **(D)** ChIP assays using anti-c-Jun antibody to detect physical interactions between c-Jun and HBD2, HBD-3 or CAMP. TLR2-or TLR4-knockdown T24 cells were infected with BCG (10 MOI for 8 h) and cross-linked with 10% formaldehyde. Expressions of AMPs were calculated as the ratio of c-Jun to Input DNA expression using densitometric analysis. All expression ratios were normalized to the untreated group.

As an innate immune response system, the TLR family recognizes pathogens and BCG activates TLR2 and TLR4 [5]. Therefore, to investigate the role of TLR2 or TLR4 in BCG-dependent AMPs release in tumor cells, we generated stable TLR2 or TLR4 knockdown lines from T24 cells and confirmed the reduced expression of TLR2 or TLR4 by Western blotting (Figure 4A, upper panel). The stable TLR2 knockdown T24 cells exhibited lower induction of phosphorylation of ERK than non-specific shRNA control cells (Figure 4A, lower panels). Whereas similar to the non-specific shRNA control T24 cells, stable TLR4 knockdown cells exhibited increase in phosphorylation of ERK by BCG treatment. Activated TLR2 and TLR4 initiate MAPK signaling pathways [21], which in turn activates c-Jun and release AMPs. As shown in Figure 4B, shRNA TLR2-knockdown T24 cells displayed reduced basal levels of phosphorylated c-Jun, which is downstream target of ERK, compared with non-specific shRNA control T24 cells. This effect was not observed in shRNA TLR4 knockdown cells (Figure 4B). We then evaluated the induced AMPs release from the supernatants of conditioned media in shRNA TLR2 and TLR4 knockdown cells. As shown in Figure 4C, BCG increased in the release of three AMPs in non-specific shRNA control T24 cells, but this effect was suppressed in only shRNA TRL2 knockdown cells. To confirm the effect of down-regulation of TLR2 on the release of AMPs due to the blockade of MEK-c-Jun downstream pathways, a ChIP assay was used to determine c-Jun binding to the HBD-2, HBD-3, or CAMP promoters. As shown in Figure 4D, shRNA TLR2 knockdown T24 cells showed reduced c-Jun binding to the HBD-2, HBD-3, and CAMP promoters, compared with non-specific shRNA control T24 cells. This effect was not observed in shTLR4 knockdown T24 cells. These data are consistent with our data showing decreased phosphorylation of ERK and c-Jun by MEK inhibitors leading to suppressed release of AMPs. These results suggest that initiation in response to BCG by TLR2, not by TLR4, is induced in a MEK-dependent manner and is responsible for the subsequent induction of the release of AMPs.

### TLR2-knockdown cells are more sensitive to BCG treatment

**Figure 5:**
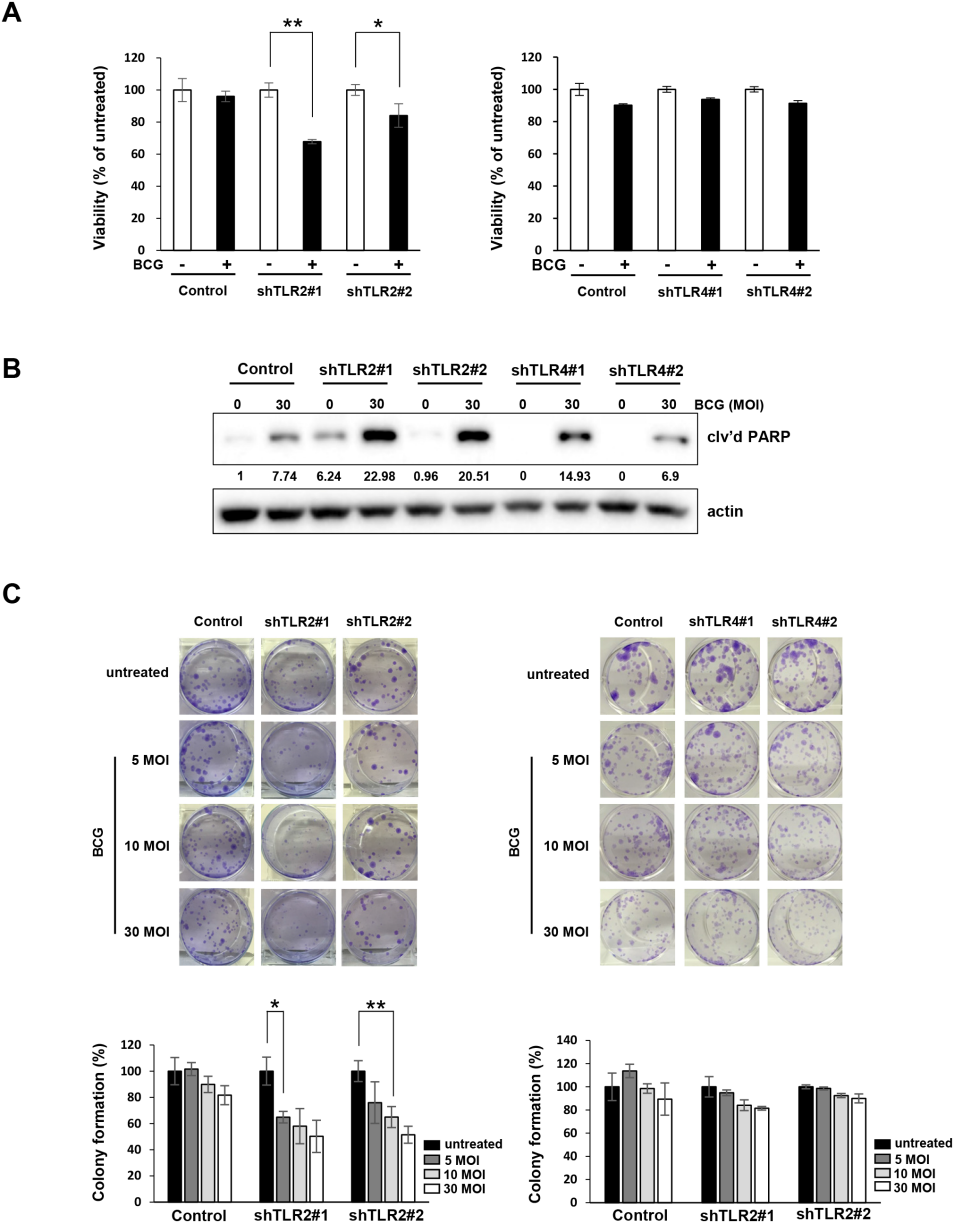
Inhibition of TLR2, but not TLR4, pathway enhances anti-proliferative effects of BCG in bladder cancer cells **(A)** TLR2- and TLR4-knockdown T24 cells were plated in 96-well plates for 24 h, and were infected with BCG (10 MOI for 48 h). Cell viability was determined using the MTT assay. Data are mean ±SD (n=6). ** *P*<0.01 and * *P*<0.05. Student's T-test **(B)** Western blotting analyses showing apoptosis by combined treatment with BCG (30 MOI) in TLR2-sable knockdown T24 cells for 72 hours. The ratio of cleaved PARP product to actin protein expression were calculated by densitometric analyses. All expression ratios were normalized to the untreated group. **(C)** Colony-formation assay of TLR2- or TLR4-stable knockdown T24 cells treated with 5 to 30 MOI BCG for 8 hours. Subsequently, cells were re-plated in 12-well plates at low density and allowed to grow for two weeks before fixation, crystal violet staining and counting. Data are mean ±SD (n=3). ** *P*<0.01 and * *P*<0.05 Student's T-test.

Finally, we confirmed that BCG inhibited the growth of TLR2-knockdown T24 cells more potently than inhibition of non-specific control cells (Figure 5A, left panel). However, in stable TLR4-knockdown T24 cells, BCG did not induce a more substantial reduction in cell proliferation compared with the vector control (Figure 5A, right panel). In addition, treatment of 30 MOI BCG highly induced cleavage of PARP in shRNA TLR2-knockdown T24 cells compared to non-specific control and shRNA TLR4-knockdown cells. BCG reduced the colony-forming ability of TLR2-knockdown cells more effectively than the non-specific control cells (Figure 5C), while this effect was not observed in TLR4-knockdown T24 cells. These results suggest that TLR2-downregulation enhances sensitivity to BCG by suppressing cell viability and colony forming ability of bladder cancer cells.

## DISCUSSION

While intravesical BCG administration has clear clinical benefits for non-muscle-invasive bladder cancer, BCG immunotherapy for treating urothelial carcinoma remains limited [22]. The precise mechanism of BCG immunotherapy, particularly downstream signaling pathways like TLR in the release of AMPs, has to be elucidated. In the present study, we demonstrated that BCG-induced MAPK signaling activation results in the release of AMPs (Figure 1A and 1B). We also demonstrated that MEK inhibitors suppressed ERK phosphorylation and decreased the viability of bladder cancer cells during BCG infection (Figures 1C and 2C). Calcium channel-stimulated *Mycobacterium tuberculosis* exhibit protective responses of activated macrophages associated with inhibited generation of reactive oxygen species (ROS) generation, which is dependent on TLR-MAPK pathways [23]. Our findings indicate that MEK inhibitors are beneficial to BCG-refractory bladder cancer cells. Furthermore, growth inhibition is elevated in MEK-inhibited BCG-infected cancer cells, and the inhibitory effects of MEK inhibitor is enhanced by inhibited release of AMPs.

To further elucidate downstream targets of MEK inhibitor-dependent AMPs down-regulation in BCG-infected cells, we analyzed c-Jun activation and binding of c-Jun, p65, and Pol II to AMP promoters during responses to BCG. Proximal promoters of AMP genes have a consensus transcription factor AP-1, and NF-κB and AP-1 are important in the regulation of AMPs in different cell types and for different stimuli [24–26]. In this study, c-Jun phosphorylation was increased after BCG-induced ERK phosphorylation (Figure 3B), which was abolished by MEK inhibition. Furthermore, MEK inhibitors obviated the recruitment of AP-1 subunit c-Jun, p65, and Pol II to AMP promoters, thereby demonstrating that the mediation of AP-1 is in part as a transcriptional factor of BCG-induced AMPs release in bladder cancer cells.

Presently, BCG induced the release of AMPs by activating ERK/c-Jun pathways in bladder cancer cells. Given the knowledge that the BCG cell wall consists of mycolic acids, arabinogalactan, and peptidoglycan, which all potentially activate TLR2 and TLR4 [5], we hypothesized that activation of TLR2 and TLR4 signaling is required in immune responses against BCG. An unexpected finding was that BCG selectively induced ERK phosphorylation and release of AMPs release only following TLR2 activation, not TLR4 activation (Figure 4A). Further analyses indicated an essential role of TLR2 for binding of c-Jun, p65, and Pol II to AMP promoters in BCG-infected bladder cancer cells (Figure 4D). The present and prior results using a reporter gene assay system and TLR knockout mice [27] suggest that purified BCG activates NF-κB promoters in a TLR2-dependent manner. TLR2 and β2 integrin triggers BCG-induced macrophage apoptosis, in which MEK/ERK activation is crucial following the engagement of TLR2 [27]. However, several reports provided evidence supporting the importance of TLR4 in BCC–induced apoptosis of macrophages [28, 29]. TLR4 is reportedly not required to control acute BCG infection, and TLR4-deficient mice display reduced bacterial clearance during long-term infection depending on the types of mutation in the common adaptor protein MyD88 [10]. Therefore, differing effects of TLR2 and/or TLR4 activation during response to BCG have been described. Presently, no significant changes were observed in the phosphorylation of ERK and c-Jun binding to AMP promoters in BCG-infected shRNA TLR4 knockdown cells in comparison with shRNA control cells, whereas a significant decrease was observed in shRNA TLR2 knockdown cells (Figure 4A and 4B). Finally, we showed that the antitumor efficacy of BCG was enhanced only in TLR2 knockdown cells compared to shRNA control cells, whereas BCG treatment had no effect on cell viability and colony formation ability in TLR4 knockdown cells (Figure 5). This efficacy was similar to ERK signaling in BCG-infected cells by MEK inhibitors. Taken together, our findings provide a rationale for BCG treatment in which the release of AMPs may be controlled by ERK phosphorylation and c-Jun activated- AP-1 transcription.

**Figure 6:**
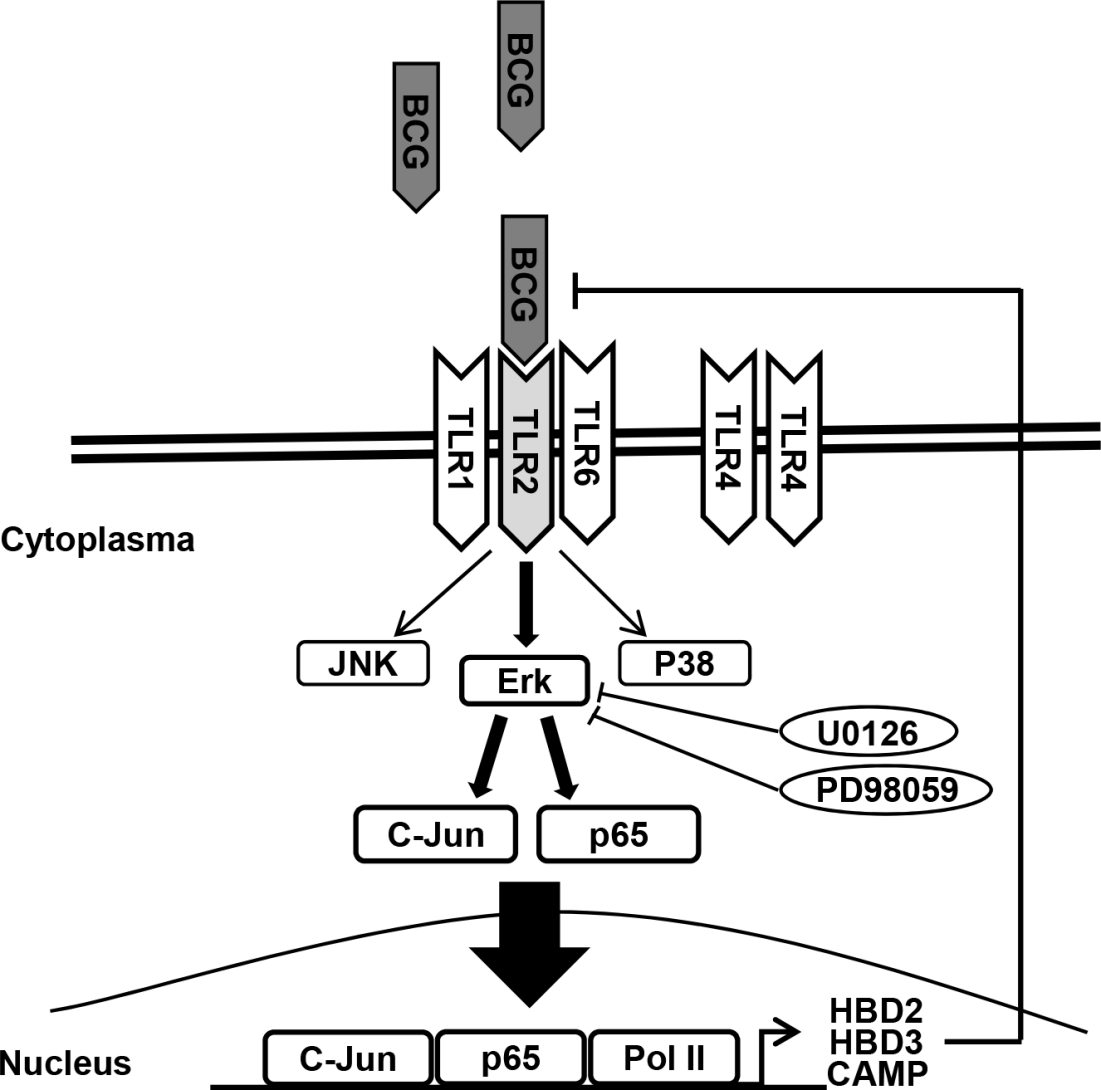
Schematic diagram of the working hypothesis BCG induces ERK phosphorylation via TLR 2, leading to c-Jun- and p65-dependent transcription of HBD2, HBD3, and CAMP. Pharmacological inhibition of ERK phosphorylation and/or genetic ablation of TLR2 enhance the anti-proliferative effects of BCG on bladder cancer cells.

In conclusion, we demonstrate for the first time that MEK inhibitors enhance sensitivity to BCG treatment in bladder cancer cells. This furthers the understanding of the underlying mechanisms blocking TLR2-derived AMPs release (Figure 6). Although MAPK signaling is implicated in the promotion of cell survival and proliferation, BCG-induced AMPs rely more heavily on TLR2-ERK signaling for the innate and adaptive immune responses. The combination of BCG plus MEK inhibitors may be useful as a salvage regimen in BCG failures. Low-dose BCG treatment may be valuable for BCG refractory bladder cancer patients.

## MATERIALS AND METHODS

### Cell cultures and reagents

The 5637 and T24 human urothelial bladder cancer cell lines were purchased from the American Type Culture Collection (VA, USA). These cell lines were confirmed to be free of mycoplasma contamination using a PCR-based detection kit (Intron Biotechnology, WA, USA). Cells were maintained in complete medium containing 10% fetal bovine serum (FBS; Gibco, MD, USA) in a humidified atmosphere with 5% CO_2_. To generate stable knockdown cell lines, plasmid TLR2 and TLR4 short-hairpin RNA (shRNA) constructs and a non-specific shRNA control were purchased from Qiagen (CA, USA). Cells were transfected with 5 μg shRNA plasmid DNA using Lipofectamine 2000 (Invitrogen, CA, USA) according to the manufacturer's instructions. To obtain stable shRNA-expressing clones, 5637 or T24 cells were plated in 6-well plates and puromycin selection (0.1–1 μg/ml) was initiated 2 days after transfection [30]. *Mycobacterium bovis* BCG was obtained as a commercial lyophilized preparation (Onco-Tice, NJ, USA). BCG was resuspended in phosphate buffered saline and aliquots with a multiplicity of infection (MOI) of 100 (1×10^7^ cells/ml) were prepared and stored at −70°C until use. MEK inhibitors U0126 and PD98069, p38 inhibitor SB203580, and the c-Jun N-termal kinase (JNK) inhibitor SP600125 were purchased from Merck Millipore (MA, USA).

### Cell viability and colony formation assays

Cells were seeded into wells of 96-well plates at 3 × 10^5^ cells per well and treated with various concentrations of rapamycin. After 48 hours, cell viability was analyzed using the 3-(4,5-dimethylthiazol-2-yl)-2,5-diphenyltetrazolium bromide (MTT) assay according to the manufacturer's instructions (Sigma-Aldrich, MO, USA). Colony formation assays were performed following treatment with BCG (5, 10, and 30 MOI) for 48 hours, at which time the cells were reseeded into 12-well plates at low density (1 × 10^3^ cells per well) in complete medium for 2 weeks [31]. Colonies were fixed with methanol and stained with 0.1% crystal violet (Sigma-Aldrich). Colony numbers were assessed visually and colonies measuring at least 50 μm were counted.

### Quantification of HBD2, HBD3, and CAMP proteins

Release of human β-defensin-2 and -3 (HBD-2 and HBD-3, respectively) and cathelicidin (CAMP) were measured by ELISA after treatment with BCG and/or MEK inhibitors in bladder cancer cells. Cells were pretreated with MAPK inhibitors. Two hours later, BCG was added and incubated for 8 hours. The conditioned medium samples were diluted and added to the recommended incubation procedures. After stopping the reaction, the samples were measured using a VERSA max microplate reader (Molecular Devices, CA, USA).

### Western blotting

Total cell lysates were resolved using sodium dodecyl sulfate-polyacrylamide gel electrophoresis and Western blotting was performed as described previously [32]. The following primary antibodies were purchased from Cell Signaling Technology (MA, USA): rabbit polyclonal antibodies against phospho-extracellular signal-regulated kinase (phospho-Erk; Thr202/Tyr204), phospho-p38 mitogen-activated protein kinase (MAPK; Thr180/Tyr182), phospho-stress-activated protein kinase (SAPK)/JNK (Thr183/Tyr185), c-Jun, p65, Pol II, cleaved PARP, and mouse monoclonal antibody against phosphorylated p70S6K (Thr389). Goat polyclonal anti-actin antibody was purchased from Santa Cruz Biotechnology (CA, USA). All experiments were performed in triplicate.

### Chromatin immunoprecipitation (ChIP)

ChIP analysis was performed using ChIP assay solutions (Santa Cruz Biotech, CA, USA) as described previously [33]. Briefly, shRNA TLR2 or TLR4 stable knockdown cells were treated with BCG for 8 h and then incubated with 10% formaldehyde to crosslink the histones to the DNA. Following preclearing, anti-c-Jun antibody, anti-p65 antibody, and anti-Pol II antibody were incubated overnight at 4°C with rotation. After elution, the crosslinks were reversed and the immunoprecipitated DNA was recovered by phenol/chloroform extraction and analyzed using PCR. The following primer pairs used for the ChIP analyses: HBD2 promoter (forward 5′-CAGGGTTTCTTCAGAACCTGA-3′ and reverse 5′-TGAGGTCTCTGGTGTCTCTC-3′), HBD3 promoter (forward 5′- CAAAGTACGGACAAGTCAGC -3′ and reverse 5′- TTCCAGCCACAGCTGCAATT -3′), CAMP promoter (forward 5′- TTGGCTTGGGAGAAGCCAT -3′ and reverse 5′- AACACAGTAGCCACCCCCAA-3′).

### Statistical analysis

The results are presented as the mean ± standard deviation (SD) for at least three separate experiments performed in triplicate. The significance of differences between values was determined by using the Student t-test. Two-sided *P*<0.05 was considered statistically significant [34].
